# Speeding up with higher quality: Introducing the new Campbell Editorial Advisory Board

**DOI:** 10.1002/cl2.1394

**Published:** 2024-03-19

**Authors:** Vivian Welch, Victoria Barbeau, Elizabeth Ghogomu

**Affiliations:** ^1^ Campbell Collaboration USA; ^2^ Bruyere Research Institute Ottawa Ontario Canada

The Campbell collaboration is the preeminent source for high quality evidence synthesis in the social sectors. Over the past 5 years that I have been editor in chief, we have doubled our publishing of systematic reviews, evidence and gap maps and methods research papers. We have also doubled our team of editors, methods editors and information specialists. However, we need to grow the community of content reviewers who provide external feedback on the domain or content of our articles.

As part of our new strategy to make evidence synthesis faster and more useful (https://www.campbellcollaboration.org/news-and-events/news/stepping-up-evidence-synthesis.html), this month, we are delighted to launch a new Editorial Advisory Board of peer referees, who are committed to contributing three to four referee assessments per year. These referees are now named on our Editorial Advisory Board page at the following link (https://onlinelibrary.wiley.com/page/journal/18911803/homepage/editorial-board). We have reached out to our networks to seek geographic and disciplinary diversity in this board. We see this peer referee board as a means to build the Campbell community, inviting new participants as well as those who are already members to continue their contributions beyond authorship. In future, we see the new Editorial Advisory Board as a pathway for people to join our other editorial activities as editors, methods editors or information specialists.

As a research‐based organization, we will monitor the effectiveness of this new Editorial Advisory Board in reducing our editorial turnaround times. These turnaround times will be publicly available next year through our journal site at Wiley online library.

If you would like to get involved in Campbell in this way, we outline our expectations for the role of these referees, available here (https://onlinelibrary.wiley.com/page/journal/18911803/homepage/referees). We invite you to get in touch by writing to info@campbellcollaboration.org. Referees are expected to have substantive content expertise in one or more social science sectors relevant to Campbell, and to be willing to review three to four Campbell articles per year. We offer recognition through Publons for peer referee contributions. For funded reviews, we can compensate peer referees for their time. We plan to launch an early career researcher network in the next 3 months, to which peer referees will be invited.

Please join us!

